# Transformation of World Health Organization’s management practice and workforce to fit the priorities of African countries

**DOI:** 10.11604/pamj.2019.34.146.19463

**Published:** 2019-11-14

**Authors:** Waogodo Joseph Cabore, Joseph Chukwudi Okeibunor, Abdulmumini Usman, Mohamed Kakay, Francis Kasolo, Raul Thomas, Francisco Katayama, Martin Matthew Okechukwu Ota, Mwelecele Ntuli Malecela, Matshidiso Moeti

**Affiliations:** 1 WHO Regional Office for Africa, Cite du Djoue, Brazzaville, Congo

**Keywords:** Core functions, functional review WHO country office workforce, health priorities

## Abstract

**Introduction:**

The WHO Regional Office for Africa developed an evidence-based tool, called the Dalberg tool to guide the functional review and restructuring of the workforce and management of the country offices to better fit the health priorities of Member States.

**Methods:**

The Dalberg tool was used in conjunction with a series of consultations and dialogues to review twenty-two countries have undergone the functional review. Results: the “core functions” in WHO country offices (WCOs) were identified. These are health coordination, strengthening of health systems, generation of evidence and strategic information management, and preparedness against health emergencies.

**Results:**

In order to standardize country office functions, categorization of countries was undertaken, based on specific criteria, such as health system performance towards Universal Health Coverage (UHC), health emergencies, burden of communicable and non-communicable diseases, subnational presence and national population size.

**Conclusion:**

Following the functional review, the staff is now better aligned with country and organizational priorities. For example, the functional review has taken into consideration: (i) the ongoing polio transition planning; (ii) the implementation of the WHO emergency programme in countries; (iii) the investment case for strengthening routine immunization in Africa; and (iv) regional flagship programmes, such as adolescent health and UHC. The delivery of the core functions above will require the hiring of additional capacities and expertise in most country offices if deemed fit-for-purpose.

## Introduction

The mandate of the World Health Organization (WHO) is to be a “directing and coordinating authority” in international health rather than a “first responder” agency [1]. Its main functions include: convening health partners; setting norms and standards; monitoring disease trends; conducting research; and providing technical and strategic support to Member States. Weak health systems in most Member States in the African Region have contributed to poor health outcomes (high maternal mortality ratios and child mortality rates, and failure to achieve the Millennium Development Goals (MDGs) and inadequate response to outbreaks and emergencies. In recent years, WHO has struggled to provide high-quality technical and strategic support to Member States, largely owing to funding challenges and also the fact that most country offices are not appropriately staffed [2]. The above issues were clearly evident during the 2014 Ebola virus disease outbreak in West Africa [3-5]. Many commentators considered WHO response to the outbreak as inadequate. Indeed, they were of the view that the organization required urgent reform [1,6]. Furthermore, the health profiles of countries vary widely within the Region. In response to some of these criticisms and challenges, the leadership of the WHO in the African Region in 2015 decided to undertake comprehensive reforms aimed at transforming the organization in the region into an accountable one capable of providing efficient and effective strategic and technical support to its Member States, through the Transformation Agenda [7-11]. Realignment of human resource capacity at regional and country levels was identified as a critical component of the Transformation Agenda [10]. The human resources of any organization are its most important assets and the caliber of people working therein is pivotal to the success or failure of the organization [2,12-20]. Schneider developed the attraction-selection-attrition (ASA) model [21] as a useful theoretical framework for understanding how the context, in terms of cultural values and structure, influences and is influenced by the set of human resource (HR) practices an organization adopts. According to ASA, as values guide judgments and decisions [21] those values and the goals of managers or decision-makers tend to become reflected in the processes, structures and culture that emerge to facilitate the achievement of organizational goals [22].

People within the organization then reflect the broader organizational values and goals [15]. The result is an “organizational logic,” or a pull toward internal consistency and a complementary relationship between the culture, vision, structure and practices of an organization, as the individuals hired to work there embody and perpetuate its core values and the practices and processes that support those values [15,23]. Four major conclusions on differentiating HR bundles could be drawn from the literature above: (a) how much is invested in HR; (b) specific focus on certain HR functions; (c) goal or priority to be achieved through HR; and (d) how the workforce is managed. Indeed, the decision of the WHO Regional Office for Africa to align its workforce with regional and country priorities and commitments is in agreement with existing theoretical perspectives on human resource management. The system's perspective highlights fitting and complementarity issues, which is arguably the most popular approach in the strategic human resource management (SHRM) literature [24-29]. Accordingly, SHRM practices must fit into one another, and the desired workforce characteristics and consequent workforce performance are achieved through the entire system of practices and the adequacy of their internal fit [30]. The relationship between SHRM practices and organizational effectiveness, from the human resource viewpoint has been amply demonstrated [31-35]. This view argues that competitive advantage is obtained when human capital resources are valuable, rare and inimitable [30,36-39]. It further argues that the critical workforce characteristics in this context are skill/ability, motivation, and resources/opportunity [23,40-42].

## Methods

**Aim and design of the functional review of WHO country offices in the African Region:** the overall objective of the functional review of WHO country offices in the African Region was to ensure alignment between the WHO country workforce and host country priorities. Four expected results were defined under the functional review as follows: (a) clear, and where necessary, revised WHO Country Office (WCO) strategic priorities, based on country needs and situation; (b) revised structure and workforce, in line with the revised WHO country priorities; (c) a financial plan, including resource mobilization to support the WCO human resource needs; and (d) improved ways of working to maximize results and staff satisfaction, and meet internal and external expectations. A key guiding principle was the joint management of the review. In other words, the restructuring was jointly led by the WHO regional and country office, with inputs from the WHO headquarters through the steering committee. The rationale for this includes: ensuring country buy-in and responsibility; consistency of the process across countries and adequate support provided to the country offices. Annex 1 highlights the four remaining principles with the rationales. Consequently, the “Dalberg evidence-based tool”, consultation tools and guidelines were developed by the Regional Office to assess alignment of workforce and operations of WHO with the host countries' health needs and priorities. The desired impact here is to have a WCO workforce that is “fit for purpose”, and which provides high quality technical advice and operational support, relevant to the host government and partners. The operational methodology of the functional review consists of establishing the structure of a country office following a multi-level consultative process with the government, United Nations agencies, funds, programmes, and bilateral and multilateral donors. This is followed by an extensive workshop with the respective country offices to enable them to align their operations and structure to country strategic priorities, while identifying the required functions and expertise. The Dalberg evidence-based tool used for staff allocation comprises a database and dashboard that, with a single-click, provides the staffing profile of a WCO with the staff requirement. It provides recommendations on target staffing for each country, as indicated in Annex 2. One of the weaknesses of this tool, however, was that it was not sensitive enough to pick the peculiar contexts and realities of the countries, beyond the epidemiological and socioeconomic profiles. In applying the Dalberg evidence-based tool therefore, there should have been more in-depth understanding of the needs of the countries, using other indicators from publicly available information (Annex 3). Scholars have argued that WHO's support to countries should be tailored to the needs and relative capacity of each country [43].

Jamison and his colleagues, in 1998, argued that support could depend on level of development. Core functions, which guide the Dalberg evidence-based staff allocation, serve all countries. On the other hand, there are other sets of supportive functions that are inversely related to the development of a country [44]. A set of consultation tools and processes were thus developed to compliment the Dalberg evidence-based tool for the functional review of the management practices and workforce of the WCOs. These tools have been employed in the review and realignment of the human resources of 22 WCOs in the Region, as of end June 2018. The process used for this functional review and realignment of the staff of the WCOs to achieve greater effectiveness in responding to country priorities and needs is presented in this paper. It is hoped that this will provide useful and instructive lessons for human resource management and organization in all other public health enterprises. Using a staff allocation model for the functional review produces a target staffing structure that realigns WHO African Region staff with country. This is because the technical expertise in a country office is dependent on the country size and need. Furthermore, the corporate services/enabling functions are determined by the number of staff and their expertise in technical categories. The staff allocation model also helps in drawing on external and internal sources to combine needs, priorities and minimum staff requirements into a target staffing. It helps in assessing country needs, using available datasets and key indicators selected with the core team of the regional office. Annex 4 demonstrates the use of the staff allocation model/tool in the functional review. It entails considerations for different classifications of staffing (fixed and variable) juxtaposed on country size. The fixed and variable staff cadres together compose the target staffing structure of the country office. However, the target staff is determined by the complexity of operations and the number of staff in the technical categories. In determining and proposing a country structure, inputs from the WHO country office, government, partners and WHO regional or global offices are considered. See the input structure in Annex 5.

### Preparatory phase

**Establishment of the steering committee:** the first step was the appointment of the oversight and steering committee of the functional review chaired by the Director for Programme Management at the Regional Office, and comprising regional cluster directors, two directors from headquarters, two WHO country representatives, the human resource and programme-budget unit managers, and the project manager of the functional review. Its terms of reference are to: oversee the process and ensuing coherence with the transformation agenda; provide direction and arbitration for the project team; ensure organization-wide consistency; and provide regular updates to the Regional Director.

**Recruitment of the project core team:** the members of the project core team were recruited to ensure smooth implementation. The composition of the project core team and their roles are as follows: project manager: manages and coordinates the work of the functional review core team, and oversees the design and implementation of new structures and change management strategy for each country; organizational design advisor: designs and implements updated organizational structures in all country offices, and provides guidance to restructuring officers for refining principles of human resource actions; career transition advisor: provides guidance on staff re-profiling and counselling throughout the implementation of the transformation strategy and the updated organizational design; project management officer: provides support in planning, implementation, monitoring and reporting of the functional review project including evaluating the work plan against key performance indicators (KPI); ensures quality results and knowledge management activities such as reports and lessons learnt; restructuring officers: responsible for liaison between the country offices and the core team. They lead the implementation of the transformation strategy in individual country offices, ensuring that the transformation strategy and new organizational design are optimized for the local context; project officers: provide support to countries in the implementation of non-human resource actions resulting from the functional review, and support the functional review team in monitoring and reporting implementation of the review recommendations.

**Development of operational guidelines:** the operational guidelines were developed as a supporting tool to guide the functional review of country offices in the Region. They were presented as steps that can be adjusted to the specific situation of each country.

### Implementation phase

**Implementation in pilot countries:** the process began as a pilot in four countries (Senegal, South Africa, South Sudan and Togo); and the feedback was positive. It involved missions to the countries and working with the WHO Country Representatives (WRs) and their team to apply the methodology developed in the guidelines. The staff allocation tool, which was developed earlier by Dalberg consultants, was used as support for the review rather than a blueprint. The staff allocation scenarios included in the tool were adapted to the specific context of each country. The approach to the review was effective, while staff members, national authorities and partners appreciated it and welcomed its timeliness.

**Internal review by the executive management and steering committee:** issues that have policy implications are brought to the attention of the Executive Management (EXM) and the Steering Committee for guidance. The EXM, comprising the Regional Director, the Director for Programme Management and cluster directors, held a one-day retreat to review the findings, identify emerging trends and lessons learnt, and provide further strategic directions for the functional review. A key output of the retreat was the endorsement of recurring expected functions as “core”. They are: support to health coordination; generation and dissemination of health information; support to health systems strengthening, including district health systems; and outbreak and emergency preparedness. Another key outcome is the harmonization of the structures in order to facilitate regional support. In line with this, all countries will have similar clustering of functions into teams, although the details of the team composition will differ, based on the specific country circumstances.

**Implementation in the second wave of countries:** a second wave of 10 countries was reviewed, taking into consideration the lessons learnt from the review of the pilot countries. The review of the second wave of countries also incorporated the outcomes of the executive management retreat.

**Independent evaluation of the functional review of WCOs:** an independent evaluation was carried out by the evaluation unit of WHO headquarters. It concluded that the functional review process was timely and well received by WRs, country office staff and other interviewees. The key issues identified by the review, which informed the recommendations made, include filling the vacant positions in the project team, increasing roles for the steering committee, more time allocated to the review process, clearer recommendations in the report content, improved timeliness of the approval process massive support for the implementation, more realistic resource mobilization assumptions and effective communication.

**Development of the final edition of the operational guidelines:** the final edition of the operational guidelines incorporates lessons learnt from the review of the pilot and the second wave of countries. Specifically, it incorporates the inputs and recommendations of the Steering Committee meetings, the Executive Management discussions, and the “mid-term assessment of the functional reviews of WHO country offices in the African Region”. The functional review exercise is organized in three steps: pre-visit works; country visit; and implementation of the recommendations. Annex 6 shows the planning phase of the country review, and highlights the various roles and responsibilities of the actors involved in the process. The three steps of the functional review developed in the operational guidelines are summarized below:

**Pre-visit works:** as a first step, the Regional Director communicates with the Minister of Health of a country to obtain a buy-in and cooperation, so as to ensure that the staff composition of the WCO is fit for the purpose of addressing the priorities of the country. Following government approval, the functional review team communicates with the WCO about the planned review. Both the review and WCO teams agree on the period for the review. Prior to the visit, the strategic health priorities and needs of the country are compiled to guide the review process, while the following key documents are provided: (a) the country cooperation strategy (CCS), which is produced by the country, in conjunction with support partners and agencies; (b) the national health strategic plan; (c) the polio transition plan; (d) the staffing plan for the world health emergencies (WHE); (e) WCO organogram; (f) programme budget; (g) donor landscape; and (h) KPI. Following the compilation and review of these documents, teleconferences are held between the functional review team at the Regional Office and the WCO, to develop terms of reference (and programme for the country mission. The terms of reference and programme of activities are shared with the staff at the WCO and the Ministry of Health. WR and Minister of Health brief relevant departments, partners and ministries on the objectives and agenda of the proposed mission. The WR then ensures that every member of the country office workforce is available and accessible to the review team during the visit.

**Country visits:** this phase consists of four separate groups of activities undertaken simultaneously during the review. The first set of activities is designed to confirm or validate country strategic directions and priorities, as contained in the background documents reviewed in Phase I. Some of the activities are: verification of the validity of the CCS; aligning priorities of the host country with those of regional and global agendas; and determining if the core functions required to meet objectives, and the expected results are clearly defined and understood in the CCS and part of global and regional priorities. It also entails developing and reviewing work plans in line with country priorities. Here, the team adopts a multi-level consultative process with the Ministry of Health and other major stakeholders. They meet with the Ministers of Health/Director-General of Ministry of Health and other related ministries such as Education, Finance and Agriculture. Additional meetings and dialogues are held with key senior staff from the respective ministries, departments and agencies. The team also meets with the United Nations Country Teams (UNCT), including the Resident Coordinator (RC) and heads of partner development agencies and bilateral bodies. Consultations also take place with civil society organizations, including the national coalition of non-governmental organizations (NGOs), NGOs working in health, such as the Red Cross, and international NGOs such as World Vision and “Médecins Sans Frontières”. This is followed by a workshop with the WCO staff to align their operations and structure with strategic priorities, while identifying required functions and expertise. The second set of actions in Phase II entails determining the financial situation of the country office. The finances, financing sources and potential for resource mobilization for the country office are all reviewed to achieve the strategic direction. The country office team is also trained in resource mobilization strategies. The third set of actions consists of reviewing the structure of the WCO human resources available to support the country. The first step here is to define the relevant structures, positions and profiles. The WCO is then guided to develop an HR action plan based on the review of the relevant organogram and positions in the HR action plan. The career counselling officer in the team also offers career counselling and holds curriculum vitae development clinics to prepare staff to compete for other job opportunities, should they be affected by any restructuring following the review exercise. Lastly, a dialogue is held on strategies for effective internal and external management of the WCO, following the restructuring exercise, so as to meet country priorities and needs.

**Implementation of recommendations:** this phase builds on the outcomes of the preceding actions taken during the preparatory and country visit phases. Based on the outcomes, an implementation plan is developed to guide the human resources unit of the WHO Regional Office and the WCO in restructuring staff to fit the purposes of the country. Restructuring the staff in countries requires significant human resource inputs and creating and revising post descriptions. It also requires the adoption of new human resource management and work practices. The various processes are monitored by the functional review team to ensure achievement of the required impact. The functional review team and Human Resources unit are also supporting countries with implementation plans, to institute the approved structures and implement the recommendations.

**Review of WHO country offices in the third wave of countries:** in planning the country visits for the third wave of countries, which started after the revision of the operational guidelines, geographical grouping of countries was considered in order to leverage the inherent potential for efficiency gains. Countries without substantive WHO country representative as well as polio high risk countries were placed at the end of the schedule for country visit.

**Ethics approval and consent to participate:** this is a routine programme activity not a research on human subject, so, we did not obtain any ethical clearance out of which we considered some lessons necessary to be placed in public domain through a scientific peer review journal.

## Results

**Results from application of the functional review model:** a total of 42 countries had been reviewed (Annex 7). In this paper, we report on the 16 out of 37 countries whose implementation plans have been approved. A number of recommendations were made for each country reviewed. So far, the overall average implementation rate of the functional review recommendations for the approved country restructuring plans ranges between 11% in the Democratic Republic of the Congo (DRC) and 98% in South Africa. The regional average implementation rate stands at 56%. Some of the key findings indicate that governments and other development partners expect WHO to do more in the areas of partner coordination, to achieve better results in the health sector and undertake hands-on capacity-building. Furthermore, they expect WHO to do more work in providing quality technical support in HSS/UHC, emergency preparedness and surveillance, health promotion, non-communicable disease prevention and control, and quality data/information management and intersectoral work. Consultations with government, health partners and country offices have led to an increase in staff, from a minimum of two in Togo to a maximum of 28 in the Democratic Republic of the Congo; while an increase in HR budget varied from a minimum of US$ 490 000 in Togo to a maximum of US$ 20 million in the Democratic Republic of the Congo. The huge increase in that country is mainly due to the fragile health systems, vulnerability to emergencies and the need to respond to the host government priorities and expectation. With regard to human resources, the first 21 restructuring plans cleared had a total of over 1,110 staff. Following the review in these countries, the technical staff increased by an average of 38% (pre-review 483 vs post-review 669) to build capacity within the ministries and partners. Fifty-nine percent of newly identified functions have been created to contribute to technical areas, while functions contributing to administrative support have decreased by an average by 17%. Contingent upon a successful resource mobilization effort at country level, international expertise is set to increase by an average of 74% (pre-review 144 to 251 post review), while national expertise increases by 37% (pre-review 323 to 443 post review). The total HR cost for the 21 country offices increased by an average 84.3% (pre-review average US$ 117 million vs post-review average US$ 216 million). Eighty-four percent average in increase in cost is attributed to technical expertise, while the remaining 16% are contributing to administrative support staff. This review exercise highlighted the need for a strong WCO with minimum capacity to support key functions needed to meet the demands of the countries. “At present, there are too many full-time job expectations with multiple skills on one person” (the WR). Some of these expectations include diplomacy/brokerage needed to achieve balance between the expectations of government and partners.

Another area of responsibility is coordination and convening of partners in health and development. Here, expectations are much higher, given the increasing number of partners. In terms of representation, the review found that there were too many UNCT, government and partner activities. With regard to resource mobilization, there was a need for sustained donor engagement, as well as clear and sustained communication. In terms of administration, with the immense pressure on national operations officers involved in high-volume transactions, there was a need for systematic oversight. Also identified were many staff issues on human relations and welfare, as were issues with the management of available resources, which needed greater alignment of skills for optimal outputs. The finding further demonstrated the need for changes in the use of working tools such as the CCS, KPI and biennial plans in the Global Management System. Areas requiring change include: (a) improvement in translation of content of the tools into “One WHO Programme” and priorities necessary for communication; and (b) elimination of the tendencies to work in silos and promote efficient mobilization of resources to contribute toward a single WHO vision. Achieving these changes would entail a systematic review of the methods used in developing the CCS and engaging the services of a focal person responsible for planning. The functional review also identified opportunities for change in the country offices, including handling the “old order” resisting a paradigm shift. In some cases, people are stuck in old thinking and are averse to change. This “resistance to change” contributes to anxieties observed while matching staff to perform new functions they are qualified for, but different from what they are used to doing. The fact that unsatisfactory matching or refusing to accept new roles may result in separation from the organization increases this anxiety. An independent mid-term evaluation team, led by the Evaluation Unit of WHO headquarters in Geneva, assessed the relevance of the steps used for the functional review. It also evaluated the extent to which the review of country offices is achieving the desired purpose. The team also identified best practices, key gaps and challenges, and provided concrete and feasible recommendations to improve the quality of future functional reviews. The result shows that practice is yielding positive results. Furthermore, the Executive Management, comprising the Regional Director, the Director for Programme Management and cluster directors, held a one-day retreat to review the findings, identify emerging trends and lessons learnt, and provide further strategic directions for the functional review. The functional review process has shown that the role that WHO is expected to play varies significantly from country to country, ranging from normative functions and technical support, to operations based on the complexities and partnership landscape. However, governments and partners in almost all countries reviewed highlighted four functions that WHO was expected to perform. A key output of the retreat was the endorsement of recurring expected functions as the “core”. These include: support to health coordination; generation and dissemination of health information; support to health systems strengthening, including district health systems; and outbreak and emergency preparedness. The remaining countries are expected to be reviewed in 2018.

## Discussion

The attainment of Universal Health Coverage, the overarching target that should facilitate achievement of all the other health targets in SDG 3, has to do directly with the performance of the health system. WHO's role in providing technical support could be further improved if adequate staff and high quality is available to provide the services as and when needed. Indeed, the health situation of women and children has led to a recommendation that WHO's work in reproductive, maternal, newborn and child health be expanded and strengthened. Scholars have argued that the context and circumstances of an organization should dictate its HR requirements [15, 23]. In line with this theoretical logic, the functional review teams identified areas where country offices needed to have their staff boosted in different categories as well as those where staffing needs to be rationalized and restructured to fit the purpose of Member States. The priorities identified by partners, country offices and the Ministry of Health varied from country to country. For example, South Sudan prioritized the availability human and financial resources and capacity for conflict management. This is not surprising given the country's conflict and emergency situation. All programmes therefore need to take this into consideration from the outset, while factoring in capacity-building and focusing on longer-term development. This is in agreement with literature highlighting the need for regular monitoring of the HR capacity for fit-to-purpose [16-20]. Most of the countries highlighted shortage of human resources as a major challenge, indicating that WHO should increase its focus on closing the gaps in this area. As a result of the review, various proposals were made to increase staff and/or cadres, which also will imply budget increases. The additional staff at the WCO also calls for supervisory staff for some positions with clusters/units in the Regional Office.

The opportunity to integrate initiatives of polio transition, WHE implementation and the UHC flagship in priority countries were also identified. Mobilization of additional resource at country level to support this increase might be challenging, in view of other competing public health issues. The long-term benefit of the impact of improved staff strength on health system strengthening and achievement of the UHC and SDG, however, make such an investment worthy and cost-effective. Nevertheless, some innovative measures to close the HR gaps could be adopted. The use of United Nations Volunteers and shared posts that are related could be cost-saving for country offices (example of project management support officers in WCO South Africa, covering Botswana, Swaziland and Lesotho). Also, functions such as communications could be sub-contracted. On the other hand, South Africa, and possibly some other countries, are the exception, since partners and the South African Ministry have identified the top priorities for WHO support in the areas of health systems strengthening, including the establishment of national health insurance, enhanced and continued HIV interventions, focus on non-communicable diseases and health promotion. The outcome of the review was that prior to the exercise, 70% of the staff could hardly play their role of furthering the priorities of the countries, in order to meet regional and global targets. Having a high proportion of misaligned staff is a recipe for inefficiency for a number of reasons. First, the workforce is likely to be pursuing goals and agendas that are incongruent with the needs of the country. Members of staff are likely to be frustrated and demotivated by the tasks assigned, being unskilled for those jobs. As illustrated in the SHRM, the WHO functional review entails ensuring a planned pattern of human resources/workforce and functional deployments and activities designed to facilitate the realization of organizational goals and objectives [5,13,14].

## Conclusion

As the priorities are met, the cost of the revision of the workforce will begin to wane. The functional review affords the organization the opportunity to take stock of its human resource profile in the context of the new demands in the countries, and in line with new regional and global targets.

### What is known about this topic

- Existing human resources management theories emphasize the attraction-selection-attrition model;
- They assume that people within the organization then reflect the broader organizational values and goals;
- These ignore the principles of inherent fit for purpose.

### What this study adds

- This paper identifies the need to regularly review human resources of an organization as the targets and goals the serve change;
- In doing this, it formulated an elaborate consultation process that ensures the restructuring of organization's human resources, based on mutual agreement, to fit the purpose of the recipients of its services;
- It demonstrated the workability of this new approach to managing the human resources of any organization.

## Competing interests

The authors declare no competing interests.
